# Supplementary material for: Identification and functional analysis of non-coding regulatory small RNA FenSr3 in Bacillus amyloliquefaciens LPB-18
Source: PeerJ. 2023 May 15;11:e15236. doi: 10.7717/peerj.15236 (PMC10194069; doi:10.7717/peerj.15236)
Supplement: Supplemental Information 4 [file peerj-11-15236-s004.zip › KO/CK-vs-T1_map/map05202.html]

KEGG PATHWAY: Transcriptional misregulation in cancer - Reference pathway


|  |  |
| --- | --- |
| **Transcriptional misregulation in cancer - Reference pathway** |  |

[
Pathway menu
| Organism menu
| Pathway entry
| Show description
| User data mapping
]

In tumor cells, genes encoding transcription factors (TFs) are often amplified, deleted, rearranged via chromosomal translocation and inversion, or subjected to point mutations that result in a gain- or loss-of- function. In hematopoietic cancers and solid tumors, the translocations and inversions increase or deregulate transcription of the oncogene. Recurrent chromosome translocations generate novel fusion oncoproteins, which are common in myeloid cancers and soft-tissue sarcomas. The fusion proteins have aberrant transcriptional function compared to their wild-type counterparts. These fusion transcription factors alter expression of target genes, and thereby result in a variety of altered cellular properties that contribute to the tumourigenic process.

|  |  |  |
| --- | --- | --- |
| Reference pathway | 184% 150% 122% 100% 82% 67% 55% | 图片下载 |
